# Reducing depression in older home care clients: design of a prospective study of a nurse-led interprofessional mental health promotion intervention

**DOI:** 10.1186/1471-2318-11-50

**Published:** 2011-08-25

**Authors:** Maureen F Markle-Reid, Carrie McAiney, Dorothy Forbes, Lehana Thabane, Maggie Gibson, Jeffrey S Hoch, Gina Browne, Thomas Peirce, Barbara Busing

**Affiliations:** 1 School of Nursing, McMaster University, Hamilton, Ontario, Canada; 2 Department of Psychiatry and Behavioural Neurosciences, McMaster University, Hamilton, Ontario, Canada; 3 Faculty of Nursing, University of Alberta, Edmonton, Alberta, Canada; 4 Department of Clinical Epidemiology and Biostatistics, McMaster University, Hamilton, Ontario, Canada; 5 St. Joseph's Healthcare, Hamilton, Ontario, Canada; 6 Veterans Care Program, Parkwood Hospital, St. Joseph's Healthcare, London, Ontario, Canada; 7 Department of Health Policy, Management and Evaluation, University of Toronto, Toronto, Ontario, Canada; 8 Hamilton Niagara Haldimand Brant Community Care Access Centre, Brantford, Ontario, Canada

**Keywords:** Depression, Ageing, Chronic Illness, Clinical Effectiveness, Home Care, Nurse-Led Interventions, Mental Health Promotion

## Abstract

**Background:**

Very little research has been conducted in the area of depression among older home care clients using personal support services. These older adults are particularly vulnerable to depression because of decreased cognition, comorbid chronic conditions, functional limitations, lack of social support, and reduced access to health services. To date, research has focused on collaborative, nurse-led depression care programs among older adults in primary care settings. Optimal management of depression among older home care clients is not currently known. The objective of this study is to evaluate the feasibility, acceptability and effectiveness of a 6-month nurse-led, interprofessional mental health promotion intervention aimed at older home care clients with depressive symptoms using personal support services.

**Methods/Design:**

This one-group pre-test post-test study aims to recruit a total of 250 long-stay (> 60 days) home care clients, 70 years or older, with depressive symptoms who are receiving personal support services through a home care program in Ontario, Canada. The nurse-led intervention is a multi-faceted 6-month program led by a Registered Nurse that involves regular home visits, monthly case conferences, and evidence-based assessment and management of depression using an interprofessional approach. The primary outcome is the change in severity of depressive symptoms from baseline to 6 months using the Centre for Epidemiological Studies in Depression Scale. Secondary outcomes include changes in the prevalence of depressive symptoms and anxiety, health-related quality of life, cognitive function, and the rate and appropriateness of depression treatment from baseline to 12 months. Changes in the costs of use of health services will be assessed from a societal perspective. Descriptive and qualitative data will be collected to examine the feasibility and acceptability of the intervention and identify barriers and facilitators to implementation.

**Discussion:**

Data collection began in May 2010 and is expected to be completed by July 2012. A collaborative nurse-led strategy may provide a feasible, acceptable and effective means for improving the health of older home care clients by improving the prevention, recognition, and management of depression in this vulnerable population. The challenges involved in designing a practical, transferable and sustainable nurse-led intervention in home care are also discussed.

**Trial Registration:**

ClinicalTrials.gov: NCT01407926

## Background

Depression affects 26% - 44% of older adults using home care services - at least twice that among older persons in general [1-3]. They also suffer from a fourfold increase in more severe forms of depression than the general population [4]. Yet, this population is one of the most undertreated populations for mental health [1,4-6]. In one study only 22% of depressed older home care clients were found to be in receipt of any antidepressant treatment, and only 15% received adequate treatment [1]. Untreated or undertreated depression in older adults is a significant public health concern, associated with greater morbidity and dependency, functional decline, diminished quality of life, poor adherence to medical treatment, increased demands on family caregivers, increased use of healthcare services and death [2,3,5,7-9]. The costs of health care associated with depression are staggering. Annual costs for depression in Canada are estimated at over 14.4 billion [10], attributable to primary care visits, hospitalization, and medication [11]. These costs are compounded by indirect costs, such as costs to family caregivers, many of whom have depression themselves.

Older home care clients using personal support services (PSS) are at particularly high risk for depression, compared to other home care clients. This segment of the population, who represent approximately 75-80% of home care users [12], are typically over 70 years of age and have co-existing chronic and acute health conditions [13], functional disabilities, cognitive impairment, or social support networks that are overextended or prone to breakdown with any shift in their health and well-being [14-16]. These are the same conditions that are associated with an increased risk of depression [2,17,18]. The magnitude of the problem has the potential to increase relative to the increasing numbers of those over 65 years of age [19], the associated increase in the prevalence of depression [17] and the increasing demand for PSS [20]. Home care is the largest component of community-based services and one of the fastest growing components of the health care system [21]. In Canada, approximately 90% of the expenditure for home care services for older adults with ongoing care needs is for PSS and 10% is for professional services [22].

Models of care that facilitate interprofessional (IP) collaborative practice are increasingly recognized as a means of addressing such demands and improving client outcomes as they lead to more efficient and effective use of health care resources and health care providers' skills. IP collaborative practice "is designed to promote the active participation of each discipline in patient care. It enhances patient and family centred goals and values, provides mechanisms for continuous communication among caregivers, optimizes staff participation in clinical decision-making within and across disciplines and fosters respect for the disciplinary contributions of all professionals"[23]. Depression generally results from an interaction of multiple and diverse risk factors, many of which are modifiable, such as persistent sleep difficulties, chronic stress associated with declining health, family or marital problems, and social isolation [17]. Studies have shown that attention to these risk factors can reduce the prevalence and severity of depression [24].

Community nurses are in an ideal position to lead an IP mental health promotion intervention to address these risk factors given their scope of practice [25,26]. A series of randomized controlled trials (RCTs) have been published on the effectiveness of various collaborative nurse-led (Registered Nurse [RN]), mental health promotion interventions among older adults with depressive symptoms in primary care [27-48] and institutional settings [49-51] in decreasing the severity of depressive symptoms [30,34,38,44,46,48-51], increasing physical functioning and health-related quality of life (HRQOL) [31,38,44,46,49], increasing mental health-related functioning and quality of life [33,34,48,49], increasing social support [50], increasing rates of depression treatment [46,48], increasing patient satisfaction with care [40,44,46], decreasing the rates of suicidal ideation [30], and decreasing costs [35,41,48].

Relatively little is known, however, about the effectiveness of a nurse-led, IP mental health promotion intervention among older home care clients with depressive symptoms who are using PSS. Other limitations of these studies include: not providing an adequate description of the nurse-led intervention and the competing alternatives; not including older adults with cognitive impairment, substance abuse, or suicidal risk; not assessing the effect of the intervention on anxiety or social support; not assessing the acceptability of the intervention; not examining which subgroups of older adults benefit most from the intervention; and not assessing the cost-effectiveness of the intervention from a societal perspective.

Nevertheless, these studies suggest that given the complex and multifactorial nature of depression, multicomponent, coordinated, and collaborative interventions provided by an IP team [52,53], using standardized screening tools and evidence-based treatment guidelines that are tailored to individual needs [52] and preferences [54], will have the greatest impact on reducing depression in this population. The benefit is even greater if the program targets individuals at risk of, suffering from, or recovering from depression [55], incorporates clinician education [17,54,56], and involves an enhanced role for the nurse (nurse care management) [32,54], regular follow-up care and a greater degree of integration between primary and specialist mental health care [32,57,58].

Despite the strength of the evidence for the effectiveness of these strategies among older adults in primary care and institutional settings, numerous challenges exist with respect to integrating these strategies into a home care setting. Many of these challenges are associated with the need for considerable reorganization of the delivery of these services for older adults with chronic needs. Specifically, home care is underfunded; in Canada, the 9% yearly growth has outpaced the 2.2% increase in spending [21,59-61], resulting in a shift in the allocation of scarce home care services away from health promotion, disease prevention, and chronic care to meet the more pressing need for post-acute care substitution [22,59,62].

The result is that home care clients at risk of, suffering from, or recovering from depression have limited access to professional services directed toward promoting mental health, especially nursing [62,63]. In many jurisdictions, eligibility for, and allocation of, home care services is directed toward addressing physical needs - mental health problems must be the secondary diagnosis in order to be eligible [63]. Other barriers to optimal depression care include inadequate collaboration and communication between home and community care providers, primary health care providers, and specialized mental health care providers, no continuity among providers, difficulties accessing specialized mental healthcare services, lack of expertise among home care providers in recognizing and managing depression [63-65], underuse of depression screening tools, competing co-morbid health conditions that mask depression [5], and stigma [3]. A final barrier is the lack of evidence-based practice standards specific to assessment and management of depression in home care for older adults [63]. Routine screening for depressive symptoms among older home care clients is not yet current practice in Canada. Thus, there is an urgent need for research to identify effective interventions to overcome these barriers to improve the prevention, recognition, and management of depression in this population.

The existing research involving home care clients is limited to descriptive, case control and quasi-experimental studies evaluating the effectiveness of nurse-led screening and referral for depression; not direct and ongoing follow-up care [3,64,66-69]. There is however, promising evidence from our previous trial, which demonstrated that a six-month nursing health promotion intervention, directed toward a general population of older home care clients (≥ 75 years) using PSS, compared with providing nursing services on demand, resulted in increased mental health functioning and related quality of life and a reduction in the severity of depressive at no additional cost, from a societal perspective [70]. Further, there is evidence from two clinical trials of the effectiveness and feasibility of RNs working with personal support workers (PSWs) in reducing depressive symptoms among older adults in long-term care settings [71,72].

The present study builds on this work by developing and testing a mental health promotion intervention involving proactive follow-up by nurse (RN) care managers working collaboratively with the PSW, the home care case manager, the client's primary care physician (PCP), and other IP home care providers (e.g., occupational therapy, physiotherapy, social work), among at-risk seniors with depressive symptoms using PSS, as opposed to the general population of seniors using home care services. The specific objective of the study is to evaluate the feasibility, acceptability, and effectiveness of a 6-month nurse-led IP mental health promotion intervention aimed at older home care clients with depressive symptoms using PSS. Our primary hypothesis is that a nurse-led IP mental health promotion intervention, delivered to older home care clients with depressive symptoms, will result in a reduction in the severity of depressive symptoms in this population. Further, we hypothesize that the nurse-led intervention will result in a reduction in depression risk factors and will pay for itself by reducing the use of expensive healthcare resources. These study findings will inform the role of nurses within the IP home care team in the management of depression and will generate lessons learned that are relevant to other home care settings. Given the increasing numbers of those over 65 years of age [19] and the increasing demand for PSS [20], this collaborative nurse-led strategy may provide a feasible, acceptable and effective means for improving the quality of life of older home care clients by improving the prevention, early recognition, and management of depression. We also discuss the issues and challenges involved in designing a practical, transferable and sustainable nurse-led strategy in home care.

Publishing the design and protocol of a study before results are available is important for several reasons. First, publication of the design of the study encourages publication of the results and informs researchers where they can find data for inclusion in a systematic review. Second, it has often been recognized that negative and adverse outcomes are less likely to be published [73]. Thus, publishing a design article can reduce the likelihood of publication bias. Finally, the publication of the design allows easier comparison between what was originally intended and hypothesized and what was actually done and provides insight into the methodological quality of a study [74].

### Research Questions

1. Does a 6-month nurse-led IP mental health promotion intervention reduce the severity of depressive symptoms among older home care clients with depressive symptoms? [Outcome evaluation]

2. Does the nurse-led intervention reduce the prevalence of depressive symptoms and anxiety, and have a favourable effect on the rate and appropriateness of depression treatment (antidepressant medication use, use of specialized mental health services), health-related quality of life, and cognitive function among older home care clients with depressive symptoms? [Outcome evaluation]

3. What are the 6 and 12 month costs of use of health services associated with the intervention? [Economic evaluation]

4. What is the feasibility of the nurse-led intervention within a defined home care setting? [Process evaluation]

5. What is the acceptability of the nurse-led intervention from the perspectives of study participants, RNs and PSWs? [Process evaluation]

The study will also provide information on the baseline prevalence, determinants and costs of depressive symptoms in older home care clients using PSS.

### Methods/Design

**Figure 1:**
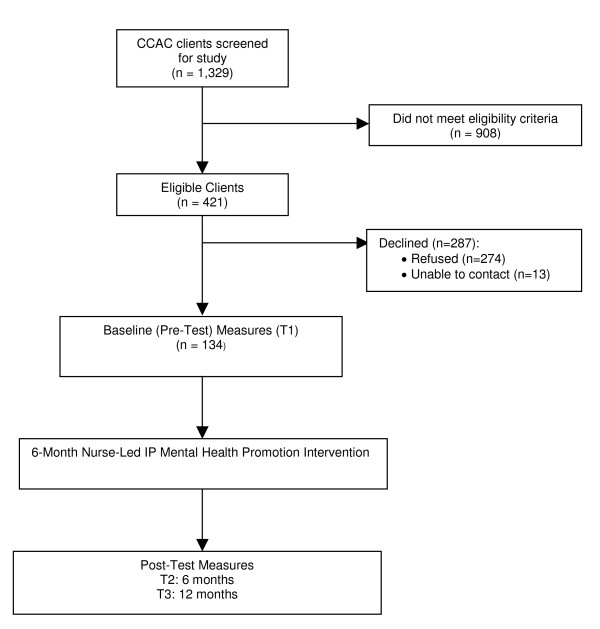
**Study Flow Diagram**.

This study is being conducted in accordance with the Tri-Council Policy Statement, "Ethical Conduct for Research Involving Humans"[75]. Ethics approval for the study was obtained from the McMaster University Research and Ethics Board and will be renewed yearly as required (#10-041). All participants provide written informed consent for participation. The methods, results, and flow of participants through the study (Figure 1) are presented according to the Strengthening the Reporting of Observational Studies in Epidemiology (STROBE) Statement: guidelines for reporting observational studies [76].

### Study Design

This is a prospective one-group pre-test post-test study that aims to recruit 250 older home care clients with depressive symptoms who are using PSS. Assessments will be made at baseline (pre-test) and after the intervention, 6 and 12 months later (post-test). Descriptive and qualitative data will be collected to examine the feasibility and acceptability of the intervention and identify barriers and facilitators to implementation.

### Participants and Setting

This study is a collaborative project between researchers at McMaster University, the University of Western Ontario, and the University of Alberta and decision-makers and practitioners in the Hamilton Niagara Haldimand Brant (HNHB) Community Care Access Centre (CCAC), Ontario Ministry of Health and Long-Term Care (MOHLTC), HNHB Local Health Integration Network, Canadian Coalition for Seniors' Mental Health, Canadian Mental Health Association, and two direct care provider agencies (Care Partners and ParaMed Home Healthcare) in Ontario, Canada. The CCAC provides publicly funded home care using a contractual model of service delivery, wherein case managers contract out home care services to agencies that provide care to clients. The HNHB CCAC is the largest CCAC in Ontario and provides home care services to the largest population of seniors in Ontario. Seniors represent 15.1% of the total HNHB population, compared to the Ontario rate of 12.9%. Approximately 15% or 30,000 of these seniors will require home care services [77]. Researchers at McMaster University and decision-makers and practitioners at the HNHB CCAC and the Ontario MOHLTC have been conducting home-care studies together since 1991[70,78,79].

Study participants are long-stay (> 60 days) home care clients, 70 years or older, newly referred to and receiving PSS through the CCAC, living in the community (not in a long-term care home), mentally competent to give informed consent (or with a substitute decision-maker available), competent in English (or with an interpreter available), not receiving palliative care services, and identified as having depressive symptoms. Recruitment procedures were developed with the goal of enrolling a heterogeneous sample of older home care clients with depressive symptoms that could be identified for the nurse-led intervention under real world practice conditions. A two-step strategy is used to recruit study participants. The first step consists of trained CCAC Case Managers identifying potential participants based on the inclusion criteria. In the second step, Case Managers contact potential participants by phone to screen them for depressive symptoms. An older person is deemed to have depressive symptoms and thus eligible for the study if he/she answers "yes" to either of the following questions: Over the last two weeks, a) have you lost interest or pleasure in doing things most of the day, more days than not? or b) have you felt down, depressed, or hopeless most of the day, more days than not? [80]. Individuals with newly detected depressive symptoms as well as those who are already receiving treatment for depressive symptoms are eligible for inclusion in the study.

The Case Manager obtains permission from eligible clients to be contacted by a Research Assistant (RA) who arranges an in-home interview to obtain their written consent, and complete the baseline (pre-test) questionnaires. To validate their informed consent to enrolling and continuing participation in the study, participants also need to score ≥ 24 on the Standardized Mini-Mental State Examination (SMMSE)[81] or have a substitute decision-maker to provide consent and complete the questionnaires on their behalf. A pamphlet is given to all participants with general study information and the contact information of the RA if they have questions or if their contact information changes before the next visit. After providing written, informed consent and completing the baseline questionnaires, participants are assigned to the nurse-led IP intervention. Figure 1 provides a summary of the process of patient selection and flow throughout the study (numbers refused, dropped, lost to follow-up, withdrawal rates, and reasons given). To date, a total of 1,329 consecutive CCAC clients have been screened for the study, and 421 (32%) have screened positive for depressive symptoms and met all eligibility criteria. In total, 134 (31.8%) of the 421 eligible home care clients have consented and entered the study.

### Development of the Intervention and Training

The intervention is an evidence-based program that was specifically designed to overcome known client, provider, and organizational system barriers to depression care in the home care setting. It was developed through a collaborative process with providers and managers from the participating agencies with the goal of integrating the intervention into standard practice once the study ends. The intervention strategy was derived from three sources: a) literature on the key features of best practice models for nurse-led IP mental health promotion interventions (discussed under Introduction), b) existing practice guideline recommendations for the prevention, recognition, and management of depression among older adults [17,18,57,58,82,83], and c) principles of problem-solving therapy (PST) using Nezu et al.'s [84] manual. A number of steps were taken to integrate purposely selected elements of these three approaches into a strategy for the care of older home care clients with depressive symptoms. Sometimes occurring simultaneously and often influencing each other, the steps included:

• Conducting an analysis of current home care practice to determine how depression is currently being addressed and to identify facilitating and limiting factors that could influence effective use of the intervention.

• Defining the goals and key features of the intervention and intervention delivery model based on empirical literature.

• Operationalizing Nezu et al.'s [84] manual for PST for use by depressed older adults with chronic conditions.

• Adopting the Home Support Exercise Program (HSEP), an evidence-based physical activity intervention for frail community-living seniors using PSWs as the primary vehicle for client instruction, motivation, and support. In the HSEP, each client is instructed on 10 simple, functional, and progressive exercises and 7 healthy eating tips, and given an illustrated booklet, a progress chart, and a short video. The HSEP exercises were designed to be simple (no equipment required), be progressive (in terms of increased time, number of repetitions, or difficulty), and address multiple areas of functioning (particularly mobility, balance, ability to transfer, and strength) [85].

• Forming an Implementation Team of providers, managers, and researchers, to systematically review existing best practice guideline recommendations, and create a depression risk management protocol. The protocol was designed to provide a systematic, standardized and evidence-based approach to the identification and modification of known risk factors for depression across disciplines. The protocol includes a summary of known risk factors for depression, standardized screening tools, and evidence-based strategies to address these risk factors (Table 1).

**Table 1:** Depression Risk Management Protocol

Risk Factor	Management Strategy	Best Practice Guideline Recommendation
Presence of Medical Illnesses Associated with Depression, e.g., Parkinson's Disease, Dementia (all types), Cardiovascular Disease, Diabetes, Stroke, TIA + Presence of Multiple Co-morbid Health Conditions + Recent major physical illness	Assessment and management of chronic and acute illness (within last 3 months); continued medical management as per physician; education for disease self-management	[17]

Taking Depressogenic Medications Taking inappropriate medications as per the Beer's criteria	Regular critical review of all medications, including herbals and OTC for depressogenic medications and potentially inappropriate medications using the Beer's criteria; Initiate *Medication Alert *to family physician for critical medication review and modification/withdrawal; Medication review by client's own pharmacist; Client education re: safe medication use, reliable means of organizing pills, medication side effects and possible interactions, inform physician/pharmacist of non-prescription medications.	[17] [57,58]

Limitations in activities of daily living	Referral to OT/PT or community resources for assistance with ADLs, e.g., personal care, meals on wheels, home maintenance services); Education of caregivers if required	[17]

Cognitive Impairment and/or recent change in mental functioning identified by SMMSE score: < 24/30 [81]	Initiate *Dementia Alert *to family physician for further assessment or discuss diagnosis and treatment; Ongoing monitoring to anticipate future needs for support; Recommend environmental adaptations; Refer to community supports; Education regarding cognitive limitations, strategies, disease process; Referral to OT/PT and family physician for treatment of perceptual disorders	[17] [57,58]

Delirium identified by the Confusion Assessment Method (CAM) [135]	Initiate *Delirium Alert *to family physician for immediate treatment Identify potential risk factors for delirium	[17] [57,58]

Anxiety identified by Generalized Anxiety Disorder (GAD-7) Screener Score ≥ 5/21 [107,108]	Provide support and information; Referral to family physician for further assessment and treatment and need for medication Initiate problem-solving therapy; Refer to community supports	[18]

Living alone Social isolation or withdrawal	Discussion of increased risk and possible change of living arrangement; Education regarding community resources to enhance social supports, e.g., Seniors club;; Participate in congregate dining; Refer to other community supports	[17] [57,58]

Excessive Alcohol Consumption (> 14 standard drinks/wk for men and > 9 standard drinks/wk for women)	Refer to community resources	[17]

Vision/hearing Impairment	Assess client for visual impairment or hearing loss; Suggest use of visual aids (glasses, magnifying glass, CNIB); Referral to audiology; Referral to CNIB	[17]

Low Income Level	Ask client if they have enough money for the things they need; able to afford necessities; Referral to Social Work or other community supports for assistance with financial matters	[17]

Low Education Level	Provide resources for literacy	[17]

Primary caregiver to a significant other with a chronic health condition Caregiver burden/strain identified by Modified Caregiver Strain Index (CSI) Score [136]	Assess caregiver health (physical and mental), level of caregiver strain, level of social contact and supports, and physical activity; Refer to community supports; Provide education regarding available community services and supports for caregiving	[17]

Adverse Life Event Chronic Stress	Ask client about any recent stressful life events (e.g., separation, losses, financial crisis, relocation to LTC); Provide education regarding ways to lessen stress; Refer to community supports	[17]

Recent Bereavement (3 to 6 months)	Refer to community supports, e.g., support groups	[17]

Chronic Pain	Assess current pain levels and treatment; Refer to family physician for review of current treatment; Refer to specialized pain clinic	[17]

Avoidant or Dependent Personality Types	When asking client about previous depression history, also ask about any other psychiatric illness such as personality disorders or anxiety	[17]

Persistent Sleep Difficulties	Assess for change in sleep patterns; Educate client about sleep hygiene techniques, and non-pharmacological approaches to improve sleep; Refer client to physician for further assessment if sleep is identified as a major issue	[17]

Nutritional risk identified by Screen II Score: < 50/64 [137]	Nutrition education; Refer to Registered Dietitian Assess need for alternative feeding methods and/or supplements; Review medications and potential for food/drug interactions	[17]

ADLs indicates activities of daily living; Screen II, Seniors in the Community: Risk Evaluation for Eating and Nutrition, version II; OT, occupational therapist; PT, physiotherapist; CNIB, Canadian National Institute for the Blind; LTC, long-term care

• Redesigning the service delivery model to support the delivery of the nurse-led intervention, which involved: a) identifying a designated team of RNs and PSWs from two existing home care provider agencies to provide the nurse-led intervention, b) developing a detailed intervention protocol, c) establishing the referral process and guidelines for communication among providers and across agencies, d) developing home care provider and client materials to support the delivery of the intervention, and e) developing role-appropriate training manuals for the RNs and PSWs to standardize the intervention.

A four-pronged approach is used to implement the nurse-led intervention. First, the investigators held separate *educational workshops *for the RNs and PSWs. Each three day workshop was supported with role-appropriate standardized training manuals and a modified version of Nezu et al.'s [84] manual for PST. The workshops focused on the scope of the issue and effective evidence-based strategies for depression prevention, detection and management among older adults. The workshops were interactive, drew on participants 'experiences, and included demonstration of skills and opportunities for practice through role plays. These workshops were supplemented by a standardized eight-hour training workshop for the PSWs in the delivery of the HSEP.

Once the workshops were completed, the nurse-led intervention was implemented using a multifaceted approach. The Implementation Team conducts scheduled *outreach visits *with the intervention providers on a monthly basis to discuss the progress of the study, provide feedback and education, discuss barriers encountered and possible solutions for identified barriers. As *reminders*, the Implementation Team periodically provides updates on the study to their staff including successes and areas for improvement related to the nurse-led strategy. At one-month intervals, the principal investigator conducts audits of the study related documentation to assess fidelity to treatment. The results of this review are used as an *audit and feedback *strategy. Audit and feedback is recommended when combined with education, outreach visits or reminders [86].

### Sample Size Calculation and Feasibility of Recruitment

The sample size was calculated to detect a clinically important difference of 3.5 points in mean change from baseline to 6 months in the primary outcome measure, severity of depressive symptoms, using the Centre for Epidemiological Studies in Depression (CES-D) Scale [87]. In the general population, a difference of this size would be associated with increased risk of functional decline, stroke, myocardial infarction and mortality [88,89]. Using a standard deviation of 9.0 as a conservative estimate and a difference of -3.5 points, a base sample size of 105 was estimated to be sufficient to address this primary outcome (two-tailed alpha = 0.05; beta = 0.20).

Our primary analysis of the factors that predict the severity of depressive symptoms will be addressed using regression analysis with the change (6-month-baseline) in the CES-D score as the dependent variable. The predictor variable: dose of the intervention (low, moderate, high), and potential confounding variables representing known risk factors for depression (female gender; prior history of depression; antidepressant medication use; living alone; cognitive impairment; family caregiver with depression; widowed, divorced or separated; low income (< $40,000 per annum); co-morbid health conditions; hospital admission in last 6 months; age; use of three or more prescription medications; presence of anxiety disorder; low social support; poor health-related quality of life; excessive consumption of alcohol; taking depressogenic medications; recent stressful life event; presence of risk factors for cardiovascular disease; and chronic pain) [17], will be included in the initial models. Norman & Streiner [90] recommend 5 participants for each independent variable included in a regression model; therefore, since the study included twenty independent confounding variables, and taking into account a possible 20% attrition rate, the minimum sample size was calculated as follows: 105 + (5 × 20) = 205 plus 20% = 246. In order to ensure a case to variable ratio that would suffice the number of variables, an even larger sample of 250 participants was planned for recruitment. The estimated attrition rate was based on an earlier trial in which 84% of the study participants completed the 6-month follow-up [70].

Our previous pilot study in the HNHB CCAC found that, on average, 100 long-stay (> 60 days) home care clients, 70 years or older, are referred to the CCAC for PSS [Unpublished Report, Markle-Reid, 2007]. Based on our previous trials with this population, it is expected that approximately 30% will screen positive for depressive symptoms [70,78,91] and meet all eligibility criteria, and 10 (33%) of these eligible clients will refuse to consent for the study. Thus, we predicted that it would be possible to prospectively recruit 250 older adults with depressive symptoms in thirteen months (20 per month). Recruitment began in May 2010 and is expected to be completed in June 2011 (13 months).

### Intervention

The nurse-led intervention consists of standard home care services plus home visitation by a designated team of RNs and a PSWs a minimum of once per month over a 6-month period. Standard home care services include routine follow-up by the CCAC case manager whose focus is on assessing the client's eligibility for in-home health services, arranging and coordinating professional (e.g., nursing, occupational therapy, physiotherapy, social work, speech-language pathology, and dietitian) and non-professional PSS, providing information and referral to community agencies, and monitoring and evaluating the plan of care on an ongoing basis through in-home assessments. The CCAC care coordinator determines eligibility and priority level for home care services and the amount and type of home care services required, based on set criteria [21].

The designated RN and PSW team provides a comprehensive, collaborative and evidence-based approach to the prevention, recognition and management of depression through regular home visits, standardized screening protocols, modification of risk factors for depression, depression education, support of antidepressant medication management, referral and linkage to health and social services, delivery of PST and social and behavioural activation, monthly case conferencing, and the development of a single, evidence-based depression management plan. The intervention, which is individualized to the client's needs, is coordinated by the RN. The RN provides leadership and coordinates communication between the client and their family caregiver, the PSW, the CCAC CM, the client's primary care physician (PCP), and other IP providers. The aim of the designated team of RNs and PSWs is to recognize, manage, and reduce the severity of depressive symptoms to enhance health-related quality of life (HRQOL) and reduce on-demand use of expensive health services. The frequency and timing of the home visits and case conferences is based on individual client needs and the results of the ongoing assessment of the client's progress toward the goals.

At each home visit, the RN systematically assesses for depression risk factors using the depression risk management protocol (Table 1), and monitors responsiveness to treatment using the Geriatric Depression Scale-15 (GDS-15) [92]. The RN supports antidepressant medication management in collaboration with clients and their PCP using evidence-based guidelines [17,18]. During the initial treatment period, when starting or modifying antidepressants, the RN conducts weekly telephone reassessment for at least four weeks to assess response, side effects and to titrate the dose [17]. Following initial improvement (< 6 on the GDS-15) [92], ongoing follow-up through monthly home visits is provided to assess clinical outcomes and support adherence to treatment [17]. For clients who do not respond to initial treatment (≥ 50% reduction in depressive symptoms is not achieved) treatment is discussed with the client's PCP [17,18]. At each visit, the PSW also monitors depressive symptoms using the behavioural rating scale for intramural psychogeriatric inpatients (GIP-28) [93]. The PSWs are expected to keep detailed records and report their observations and concerns to the RN. Two RCTs have demonstrated that use of the GIP-28 by PSWs working in collaboration with an RN is an effective, feasible, and acceptable strategy for increasing the recognition of depressive symptoms in older adults [71,72]. Clients and their caregivers also receive education on depression using printed educational materials (from the Canadian Coalition for Seniors' Mental Health and Canadian Mental Health Association).

Each client's treatment regimen is discussed by the RN and PSW at a case conference held a minimum of once per month for 6 months. A depression care booklet is used to systematically guide the RN and PSW team through a series of questions that triggers assessment of depressive symptoms, current treatment, treatment response, risk factors for depression [17,18,57,58,82,83], use of social and behavioural activation and PST, and recommended actions for reducing depressive symptoms and promoting health-related quality of life.

The RN assesses client's problem-solving strengths and limitations using the Problem-Solving test, and provides a minimum of 6 sessions of PST using Nezu et al.'s [84] manual, as part of their home visit. PST consists of teaching participants a five-step problem-solving model. The overall goal of PST is to help the depressed person develop skills in order to approach life problems in an active fashion, using the steps of problem-solving as a coping strategy [17,84]. A series of RCTs conducted in primary health care and other community settings have demonstrated that PST provided by RNs is an effective, feasible, and acceptable treatment for depression in older adults [40,41,46,49,94,95]. We selected Nezu et al.'s [84] problem-solving method because of its sequenced approach to teaching problem-solving and its emphasis on problem orientation, a critical element of problem-solving therapy [96].

In our application, we use the therapy's five steps which consist of: a) developing a positive, optimistic attitude toward the problem and one's ability to cope with it; b) defining the problem and setting realistic goals; c) discussing and evaluating different ways to reach goals; d) creating action plans; and e) evaluating their effectiveness in reaching goals [84]. However, the RN is more directive than specified in the original PST instructions, and participants are encouraged to focus on less complex problems to facilitate learning. During the initial home visits, clients are asked to identify the most frequent or stressful problems they are encountering and how they are managing the problem. The RN then assists the client to effectively apply the problem-solving steps to address each problem in a sequential order as prioritized by the client using a problem-solving worksheet.

Social and behavioural activation involves assisting and encouraging clients to participate in a regular physical activity program that is tailored to individual needs by providing information, personal counselling, support, and skills training to overcome barriers to increasing their physical activity levels. Physical activity is encouraged through the use of the Home Support Exercise Program (HSEP) [85]. The RN introduces the client to the program, assesses their interest and ability, and authorizes the PSW to start the client on the program. Using one-on-one coaching, the PSW and client begin working through the ten exercises and seven healthy eating tips with the client, along with the HSEP resources and a progress chart [85]. The goal of social and behavioural activation is to increase client and caregivers' interactions outside the home by facilitating referrals to seniors' programs, support groups or other community services [49].

**Figure 2:**
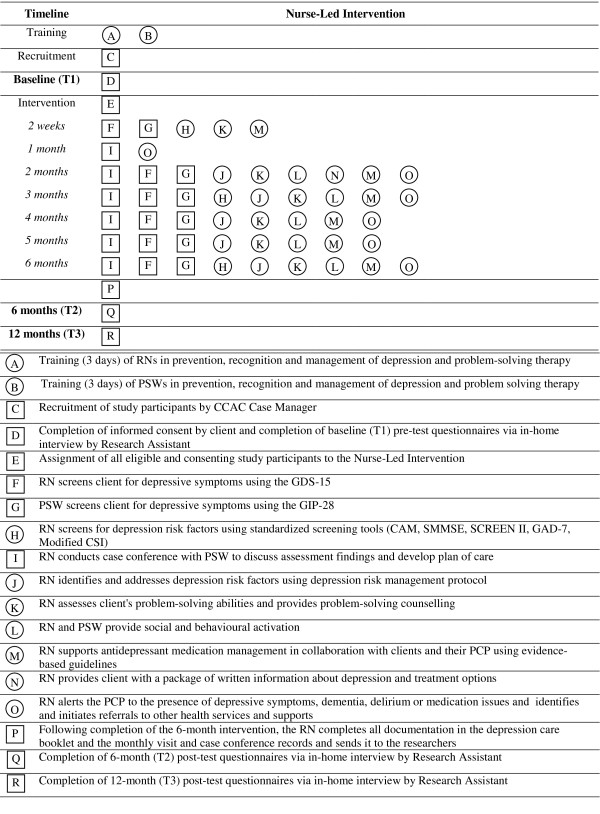
**Graphical Depiction of Intervention and Measurements**. Squares represent fixed elements. Circles represent activities that are flexible. Measurements are bolded. This graphical method was proposed by Perera et al. [134].

The RN develops an evidence-based plan of care to meet mutually agreed upon and attainable goals in collaboration with the client and caregiver, the PSW, the client's PCP, and the other members of the IP team. The plan includes specific short-term and 6-month goals, a list of actions and referrals and a record of all recommendations. The results of the initial and ongoing assessment, and the client's response to treatment, are documented in the care plan and reviewed during each case conference. This includes documenting the client's level of adherence to the recommendations and reasons for non-adherence. The RN liaises with the client's PCP and other providers to initiate referrals to a comprehensive range of services and supports to address individual client needs. The RN alerts the PCP to the presence of depression, dementia, delirium using a standard letter, which is sent directly to the PCP requesting further assessment and treatment (Figure 2).

### Outcome Evaluation

The aim of the outcome evaluation is to examine the effect of the IP mental health promotion intervention on clinical outcomes among the target population.

#### Variables and Measures

Independent interviewers, blinded to the purpose of the study, assess participants at baseline (pre-test), and after the intervention (6 months) and again at 12 months (post-test) through a structured in-home interview lasting about one hour. Previous research suggests that 6-months is an optimal time to assess the immediate effects [32,97] while minimizing attrition rates [98]. A follow-up assessment at 12-months will establish whether any significant change is maintained 6 months after the intervention has taken place (see Figure 2). Three interviewers, who are Registered Nurses with previous experience working in community-based settings, were trained in consent and data collection procedures. Training included orientation manuals, practice sessions, and inter-rater reliability testing. Overall agreement for categorical variables was very good (Kappa statistic = 0.80, CI:95% 0.76, 0.84) as was overall agreement for continuous variables (ICC = 0.96, CI: 95% 0.92, 0.99) [90]. Table 2 provides an overview of all outcome variables and measures used in the study.

**Table 2:** Variables and Measures

	Variables	Measures	Timing of Data Collection
**Participant Characteristics**	Age, Gender, Medical Diagnoses, History of Depression, Culture, Informal supports, Education, Living arrangement, Income, Marital status, Use of prescription medications, Recent stressful life event, Alcohol Use, Sleep Pattern	Sociodemographic Questionnaire	T_1_

**Outcome Evaluation**	Depressive Symptoms	Centre for Epidemiological Studies in Depression Scale (CES-D) [87]	T_1_, T_2 _and T_3_

	Anxiety	Generalized Anxiety Disorder Screener (GAD-7) Scale [107]	T_1_, T_2 _and T_3_

	Health-Related Quality of Life	SF-12v2 Health Survey [109]	T_1_, T_2 _and T_3_

	Cognitive Impairment	Standardized Mini-Mental State Examination (SMMSE) [81]	T_1_, T_2 _and T_3_

	Depression Treatment: Antidepressant Medication Use Use of Specialized Mental Health Services	Health and Social Services Utilization Inventory (HSSUI) [Unpublished Paper, Browne, Gafni & Roberts, 2006]	T_1_, T_2 _and T_3_

**Economic Evaluation**	Health Services Utilization, from a Societal Perspective	HSSUI [Unpublished Paper, Browne, Gafni & Roberts, 2006] CCAC Records	T_1_, T_2 _and T_3_

**Process Evaluation:** ***a)Feasibility of the Intervention***	Recruitment Rate and Representativeness of Sample	CCAC Records	T_1_

	Number and Duration of Home Visits and Case Conferences	Monthly Visit and Case Conference Record	T_2_

	Fidelity to Treatment	Fidelity Scale	T_2_

***b)Acceptability of the Intervention***	Engagement Rate	Monthly Visit and Case Conference Record	T_2_

	Level of Adherence to Health Care Provider Recommendations	Client Care Plan	T_2_

	Perceptions of Intervention by Study Participants	Semi-Structured Interview	T_3_

	Perceptions of Intervention by Intervention Providers	Focus Group Interviews	6 and 18 months after initiation of the intervention

T_1_: Baseline; T_2_: 6 months after baseline measures; T_3_: 12 months after baseline measures

*The primary outcome *is the change in severity of depressive symptoms from baseline to 6 months as measured by the CES-D score [87]. The CES-D has been used in prior studies of older adults with mood disorders [99,100], and has a high degree of reliability [101], content, construct and criterion related validity [102], distinguishes between depressed and non-depressed people [103], and is a sensitive tool for measuring changes in depressive symptoms over time in psychiatric populations [104]. Previous research on the CES-D involving older adults yielded sensitivity of 92% and a specificity of 87% when compared to the Structured Clinical Interview for the DSM-III-R [105,106] by using a cut-off point of 21 to distinguish older people with and without depressive symptoms.

*Secondary outcomes *include changes in the following variables from baseline to 6 and 12 months: a) prevalence of depressive symptoms using a cut-off score of ≥ 21 on the CES-D [87], b) severity of anxiety measured by the Generalized Anxiety Disorder Screener (GAD-7) scale [107], c) prevalence of anxiety using a cut-off score of > 5 on the GAD-7 scale [108], d) rate and appropriateness of depression treatment (antidepressant medication use, use of psychiatrist or other specialized mental health services) measured by the Health and Social Services Utilization Inventory (HSSUI) [Unpublished Paper, Browne, Gafni & Roberts, 2006], e) health-related quality of life measured by the SF-12v2 health survey [109], and f) cognitive impairment using a cut-off score of ≥ 24 on the SMMSE [81]. These outcome measures were selected on the basis of their length, low level of burden, ease of administration, and reliability and validity in our previous homecare trials [70,78,91].

#### Data Analyses

Quantitative data will be cleaned, checked for out of range values, skip pattern problems and duplicates. Tests of normality will be completed to determine appropriateness of statistical methods. All analyses will be performed using the Statistical Package for the Social Sciences (SPSS) version 19.0 for Windows on an intention-to-treat basis. All statistical tests will be performed using two-sided tests at the 0.05 level of significance. For all models, the results will be expressed as effect (or odds ratio for binary outcomes), standard errors, corresponding two-sided 95% confidence intervals (CI), and associated p-values. Descriptive analysis of demographic characteristics will be expressed as a mean (standard deviation [SD]) or median (minimum-maximum) for continuous variables and count (percent) for categorical variables.

The primary analysis to address the main outcome measure (change in mean scores from baseline to 6-months in the severity of depressive symptoms) will be tested using analysis of covariance (with pre-intervention scores as covariates). The factors that predict the severity of depressive symptoms will be addressed using regression analysis with the change (6-month-baseline) in the CES-D score as the dependent variable. The predictor variable dose of the intervention (low, moderate, high), and potential confounding variables representing known risk factors for depression, will be included in the initial models. Model assumptions and goodness-of-fit will be assessed by examining the residuals for model assumptions and chi-squared test of goodness-of-fit or qqplots and Hosmer-Lemeshov tests for logistic regression models. We will assess multicollinearity by investigating associations among the confounding variables. For linear models, we will use the variate inflation factor (VIF) to assess collinearity. Variables having a VIF greater than 10 will be considered collinear and will be excluded from the analysis [110]. The secondary hypothesis of change in the prevalence of depressive symptoms, anxiety, HRQOL, cognitive impairment, and the rate of depression treatment from baseline to 6 and 12 months, will be tested using multiple regression (or logistic regression for binary outcomes) using the same approach as in the primary analysis. The possibility of co-interventions occurring such as mental health-related counselling and support programs will also be monitored.

We will also perform the following sensitivity analyses which will involve: a) use of multiple imputation to handle missing data, b) analysis of all outcomes analysed simultaneously using multivariate analysis of covariance (MANCOVA) to account for correlation among them, c) longitudinal analysis of data using generalized estimating equations (GEE) to account for the serial correlation of all outcomes at baseline, 6 and 12 months within a subject, and d) clustering among individuals who live together. The GEE is a technique that specifies the correlation structure between participants [111]. This approach produces unbiased estimates under the assumption that missing observations will be missing at random. An amended approach of weighted GEE will be employed if missingness is found not to be at random [111]. We will assume an autoregressive AR(1) correlation structure for the GEE analysis, which assumes that serial responses measured closer to each other are more correlated than those that are far apart.

### Economic Evaluation

The aim of the economic evaluation is to determine the 6 and 12 month costs of use of health services associated with the intervention from a societal perspective, where all costs are assessed regardless of who bears them. A societal perspective is recommended for studies relevant to societal policy decisions [112].

#### Variables and Measures

The *costs of use of all types of health services *from baseline to 6 and 12 months will be determined using the HSSUI [Unpublished Paper, Browne, Gafni & Roberts, 2006]. The HSSUI consists of questions about the respondent's use of six categories of direct health care services: a) primary care, b) emergency department and specialists, c) hospital days, d) seven types of other health and social professionals, e) medications, and f) lab services. Inquiries were restricted to the reliable duration of recall: 6 months for remembering a hospitalization and a visit to the physician, and four days for use of a prescription medication. The 6-month cost data will be derived from "quantity" data reported on the HSSUI and 2009-2012 "price" data obtained by our team for the HSSUI [Unpublished Paper, Browne, Gafni & Roberts, 2006]. The product of the number of units of service (quantity) and unit cost (price) is total cost. The six month estimate can be multiplied by two to approximate the total health service utilization cost for the 12 month follow-up period. The HSSUI has been previously tested and assessed for reliability and validity [113,114] and is acknowledged as one of the few published measures of ambulatory utilization that is empirically validated [115]. The costs of use of health services measured by the HSSUI will also include the costs associated with the delivery of the nurse-led intervention. Administrative data on the use of CCAC services will be used when applicable to validate the self-reported data [116]. The HSSUI will be incorporated into the structured in-home interview at baseline, 6 and 12 months.

#### Data Analyses

We will conduct cost-effectiveness analysis (CEA) on person level cost and effect data using net benefit regression [117,118]. The dependent variable for the net benefit regression in this analysis will be *nb *which equals *effect *· $ - *cost *where *effect *= improvement in CES-D, $ = the monetary value of each unit of *effect *and *cost *= the costs accrued over the study period. Different values of $ will be used to explore how sensitive the results are to assumptions about $. In the base case scenario, the dependent variable *nb *will be modelled as a function of the patient variables described above and a stochastic error term (ε). We will estimate the coefficient β_0 _in the regression nb = β_0 _+ **β_x _x **+ ε where **x **is a vector of patient variables that have been centred on their means, **β_x _**is a vector of their respective coefficient estimates, ε is a stochastic error term and β_0 _is an estimate of cost-effectiveness. When β_0 _> 0, the intervention is deemed to be cost-effective since the value of the extra benefit is greater than the extra cost [117]. Net benefit regressions will be run for a variety of $ values and then the probability of cost-effectiveness will be plotted against values for $ on a cost-effectiveness acceptability curve [119]. In this way, the research findings will allow different decision makers to use different preferred values of $. By placing the CEA in a regression framework, we will be able to use a wide variety of regression tools while exploring key patient characteristics and using regression model diagnostic tools [120].

### Process Evaluation

The purpose of the process evaluation is to determine the feasibility and acceptability of the intervention. Assessment of feasibility determines whether the intervention can be successfully implemented to establish actual exposure to the intervention as it was intended, and to examine which components of the intervention were successful and which ones were not [121]. Assessment of acceptability determines the suitability of the intervention from the perspective of the study participants and the intervention providers [121]. Table 2 provides an overview of all process variables and measures used in the study.

#### Variables and Measures

Assessment of the *feasibility of the intervention *will include: a) the reach of the intervention, defined as the proportion of the intended target population that actually participated in the intervention, b) the dose delivered, defined as the number and duration of the home visits and case conferences during the 6-month intervention, and c) the level of fidelity to treatment (the extent to which the RNs and PSWs adhere to the components of the intervention). We are keeping a record of all clients who meet the eligibility criteria for the study to determine the recruitment rate and assess the reach of the intervention. Alphanumeric characteristics (age, gender, medical diagnosis, referral source) of the clients who are assessed as eligible for the study but refuse to participate will be collected at baseline to assess the representativeness of the sample. Data on the dose of the intervention and the RNs' and PSWs' actual adherence to the intervention will be gathered by the RNs and PSWs themselves by means of prospective self-reports. The RNs and PSWs will use the monthly visit and case conference record and the depression care booklet after each case conference to record which components of the intervention were applied. Each item will be scored as a done/not done binary variable (see Table 3 for the key intervention features that will be scored) [122,123].

**Table 3:** Fidelity Scale

Intervention Components	Data Source	Yes	No
**Staffing and Supervision**			

RNs and PSWs receive standardized training	Attendance record		

RNs and PSWs meet with investigators on a monthly basis	Attendance record		

**Follow-Up**			

In-home visits by trained RN at least once per month for 6 months	Monthly visit records		

In-home visits by trained PSW at least once per month for 6 months	Monthly visit records		

**Standardized Screening Tools**			

Depressive symptoms monitored once per month using the GDS-15 [92]	Depression care booklet		

Cognitive status monitored at baseline, 3 and 6 months using the SMMSE [81]	Depression care booklet		

Presence of delirium monitored at baseline, 3 and 6 months using the CAM [135]	Depression care booklet		

Anxiety monitored at baseline, 3 and 6 months using the GAD-7 [107]	Depression care booklet		

Nutritional status monitored at baseline, 3 and 6 months using the SCREEN II [137]	Depression care booklet		

Caregiver stress monitored at baseline, 3 and 6 months using the Modified CSI [136]	Depression care booklet		

Behavioural problems due to cognitive problems and mood disorders monitored once per month by PSW using the GIP-28 [93]	Depression care booklet		

Problem-solving ability monitored at baseline, 3 and 6 months using the problem-solving test [84]	Depression care booklet		

**Depression Education**			

Received education about depression and treatment options using printed educational materials	Depression care booklet		

**Interprofessional Care**			

Participants are discussed at a case conference at least once per month for 6 months	Monthly case conference record		

Referred to health and social services, as needed	Depression care booklet		

Interprofessional client service plan developed	Client service plan		

Evidence of communication between RN, PCP and other IP providers	Depression care booklet Client service plan		

**Treatment Planning and Delivery**			

Problem-solving therapy delivered at least once per month for 6 months	Depression care booklet		

Social and behavioural activation provided by RN and PSW	Depression care booklet		

Antidepressant medication management is provided using evidence-based guidelines	Depression care booklet Client service plan		

Assessment of the *acceptability of the intervention *will include: a) the level of engagement to the intervention (the number of participants who receive at least one home visit over the 6-month period), b) perceptions of the intervention by study participants as measured by semi-structured interviews, and c) perceptions of the intervention by the intervention providers as measured by focus group interviews. Data on the perceptions of the intervention by study participants will be determined through the use of five open-ended questions during the final 12-month interview: "Which aspects of the nurse-led program were most helpful to you?" "Which aspects of the nurse-led program were the least helpful to you?" "Which aspects of the nurse-led program have you continued to use?" "Which aspects of the nurse-led program haven't you continued to use?" "Would you recommend this program to other people?" The RNs and PSWs will be invited to a focus group interview at 6 and 18 months following initiation of the intervention to discuss their perceptions of the intervention, including the intervention components they perceive to be most and least helpful and to identify barriers and facilitators to implementation: "What aspects of the nurse-led intervention have worked well for clients and their families, home care providers, and the organization and system?" "What kinds of challenges have there been with respect to implementing the nurse-led intervention for clients and their families, home care providers, and the organization and system?"

#### Data Analyses

Study participants will be compared with clients who are assessed as eligible for the study but refuse to participate on their alphanumeric characteristics at baseline using independent t-tests for continuous variables and chi square for categorical variables. Descriptive analysis of the dose of the intervention will be expressed as the mean (standard deviation [SD]) and range (minimum-maximum) number of home visits and case conferences. Data on the RNs' and PSWs' actual adherence to the intervention will be categorized as the proportion of times each component of the intervention was implemented. Content analysis of the older home care clients' responses to the open-ended questions will be used to analyse the qualitative data. Participant answers will be sorted into categories and themes. Descriptive analysis will be performed for the frequency of the answers for each question.

The focus groups will be digitally recorded and transcribed verbatim by an experienced transcriptionist and checked for accuracy by a Research Assistant. Qualitative data will be managed using N-VIVO 8 software. Three of the investigators (MMR, CM, DF) will systematically review all transcripts and inductively generate a list of codes by hand describing themes. The codes will be grouped into themes (a higher conceptual level) and sub-themes. The investigators will use a constant comparison approach to interpret data. This will involve reviewing coded data that supported themes and continually referring to previously coded sections for comparison. By comparing and contrasting the coded data, sub-themes, themes, interrelationships and patterns will be revealed. Differences of opinion will be discussed until agreement is reached.

## Discussion

In this paper we describe the background, design and methods of an ongoing prospective study of the effectiveness of a nurse-led, IP mental health promotion intervention aimed at older home care clients with depressive symptoms. This research has two important innovative aspects. First, this is the first study that investigates the effectiveness of a 6-month nurse-led, IP mental health promotion intervention among this population. Studies on the effectiveness of nurse-led mental health promotion interventions in home care have involved short-term screening and referral for depression rather than ongoing treatment and regular follow-up care over a 6-month period. Second, it is the first study to investigate the effectiveness of such an intervention among at-risk seniors with depressive symptoms using PSS, as opposed to the general population of seniors using home care services. Such individuals are considerably frailer than the general population of seniors receiving home care services and are often excluded from community-based studies. Thus, this study will make an important contribution by providing knowledge of the effectiveness of a nurse-led mental health promotion intervention among a more vulnerable group of older home care clients than recruited in previous studies. Stratifying individuals into different levels of risk is important from a clinical and economic perspective because those at greatest risk are more likely to benefit from preventive efforts and can be targeted specifically [17,37].

Our primary hypothesis is that a nurse-led IP mental health intervention offered to older home care clients with depressive symptoms, will result in a reduction in the severity of depressive symptoms, and will pay for itself because the cost of the health care resources related to the intervention will be offset by a lower cost of use of other expensive health care resources. For example, in Canada, hospital costs constitute the largest component of health care expenditures for depression, at approximately $3.8 billion dollars per year [10]. An older person with depression has a 25% chance of being hospitalized within one year, with the average acute care cost of treatment of about $21,800 per person with depression [124]. Based on previous research, we expect to decrease the average use of acute hospitalization by 17% in the 250 participants receiving the nurse-led approach [69], which translates into a cost savings of 43 people × $21,800 = $937,400 in the same year due to prevention of acute hospitalization for depression; this by itself should create enough savings to pay for the intervention. However, to determine if our hypothesis is correct, we will examine costs expended as well as costs averted by all sectors of society (e.g., beyond acute care hospital costs), to reflect a societal perspective.

If our hypothesis is correct, we will conclude that a nurse-led IP mental health promotion intervention, proactively provided to older home care clients with depressive symptoms, reduces depressive symptoms and enhances health-related quality of life at no more cost to society as a whole, thus making the intervention highly feasible given its clinical benefits. The results of this project will provide evidence for the feasibility, acceptability, and effectiveness of an innovative model of service delivery that has the potential to significantly improve the quality of life of older home care clients with depressive symptoms and reduce demand for health services achieved by: improving the recognition and management of depressive symptoms; linking home care, primary healthcare, and specialized mental health services; facilitating effective IP collaboration and teamwork; facilitating timely access to depression-related care and services; promoting adoption of best practice guideline recommendations for depression care; providing regular follow-up care and treatment over 6 months; developing and implementing standardized prevention, education, and screening protocols; and expanding health care provider knowledge and skills in depression care, chronic disease management, and IP collaboration. The results of this research will add to the accumulating evidence that early, proactive, and comprehensive care for older adults with chronic needs is both more effective and no more expensive in a system of national health insurance than providing services on a limited, reactive, and piecemeal basis [70,78,91,113,114].

The results will also provide information about the processes and strategies that foster: effective IP collaboration, use of best practice depression guideline recommendations, and integration and coordination of home care, primary healthcare, and depression-related services for this population. The knowledge gained from this research will be made available to decision-makers and clinicians to inform policy and practice related to the allocation and delivery of services for the prevention, early identification, and management of depression among older home care clients. These findings will have application in other home care and community-based practice settings.

Many older adults using PSS are "at-risk" for depression, but the rate of depression and depression risk factors have not been previously investigated in this population. Our results will provide information on the prevalence, determinants and costs of depressive symptoms among older home care clients using PSS. On the basis of previous research among a general population of older home care clients [1,27,70], we expect that depression will be highly prevalent in this population and associated with poor health-related quality of life, low social support, and higher use of health services. We also hypothesize that depression in this population will be largely untreated or undertreated [1]. The two step screening and recruitment process may identify seniors who would not in the normal course of events have received any treatment for their depression, thus highlighting the importance of incorporating screening into routine clinical practice. Improving understanding of the prevalence of depression will help to raise awareness of the important role of home care in recognizing, managing, and reducing depression in this population. Knowledge about risk factors for depression among older adults using PSS, can be used to guide the allocation of scarce home care resources to those most likely to benefit from preventive efforts.

Several limitations to this study should be noted. First, the single study site may limit generalizability of our findings. Second, there is no comparison group in the one group pre-test post-test design. Third, sampling bias may influence the results, as those who volunteer to participate may have been more likely to be receptive to the intervention. We will assess the extent of non-response bias by comparing the characteristics of study participants to those who decline participation in the study on their alphanumeric characteristics at baseline. In addition, those eligible clients who refuse to enter the study will be asked about their reasons for refusing to take part in the study. Content analysis of these factors will be conducted to provide feedback to guide refinement of the intervention to make it suitable to those who did not participate. Fourth, clinical depression was not evaluated in this study; in future studies, it would be important to include a structured clinical interview to confirm a DSM-IV based diagnosis of major or minor depression versus depressive symptoms. Lastly, the use of a proxy respondent as a source of data for the study participants with limitations in cognition, physical health or language, may result in either an overestimation or an underestimation of the results [125].

The design of an effective real-world model for implementing a nurse-led IP mental health promotion intervention that can be implemented in clinical practice, reach the target population, be effective across diverse providers and settings, and be able to be maintained over time [126], will require attention to four key challenges that will be addressed in this study. The first challenge centres on the need to reach those older home care clients most likely to benefit from the nurse-led intervention. Our inclusion criteria will identify older adults who are at risk of developing depression and thus, most likely to benefit from the intervention. It is expected that through the use of a minimal or less restrictive set of selection criteria, we will increase the heterogeneity of our sample. For example, we will include older home care clients who have co-morbid medical conditions and dementia that have typically been excluded from community-based trials. This heterogeneity will improve the representativeness of the sample, so that the variability in older home care clients seen in everyday practice is reflected thereby, enhancing the generalizability and clinical applicability of the research findings [127].

Subgroup analysis is planned to examine the influence of participant characteristics on the outcomes. The analysis is intended to investigate who with what characteristics benefits most at what expense, from the intervention. This will be accomplished in two steps. In the first step, the pattern of change in the outcome level is estimated for each participant. In the second step, inter-individual differences in the pattern of change will be examined in relation to participant characteristics and receipt of the intervention [127]. The results can be used to inform implementation of the program in other settings by enabling home care agencies to tailor the intervention to individual needs and to target scarce resources to those most likely to benefit from the intervention.

The problems related to recruitment and retention of older adults in research are well documented [128]. To address recruitment barriers, we utilize clear but simple communication of study procedures, risks, and potential benefits; we give the client the time needed to decide; we have a clear protocol for contacting potential participants and flexible scheduling; and we educate unpaid caregivers about the study (with the client's agreement) if the client wants extra support, even if the patient is able to make a decision for themselves. Tymchuk and Ouslander [129] wrote of the need to orient or prepare the client to receive information from the researcher prior to the actual explanation of the research. In this study, this stage is considered vital and involves determining if the client has any cognitive or communication difficulties. Awareness of any communication difficulties or cognitive limitations prior to the initial contact enables action to address and compensate for these challenges from the outset.

To enhance retention, interviewers build rapport and trust with the participants; the study coordinator uses a participant-tracking plan and the interviewers maintain between-assessment contacts with participants [130,131]. A week before each interview, a reminder letter is mailed to the client's home. Three days before the scheduled visit, the interviewer phones to remind participants of the data collection visit. Participants are also compensated for their time ($15 after the baseline interview and $10 for the 6-month interview). On a monthly basis, Case Manager recruiters and interviewers meet with the principal investigator to discuss recruitment and data collection procedures. These meetings have been instrumental in identifying recruitment and data collection problems. It has resulted in clarification of inclusion criteria, data collection procedures, and suggestions for additional strategies for recruitment and retention.

The second challenge centres on the need to assess the feasibility of the intervention within the local context. It is expected and will be accepted that the intervention protocol may require some adaptation to accommodate client's needs and the unique characteristics of the local context, which will result in variation in the intervention components and dose to which participants are actually exposed [127]. It is therefore important that the processes and outcomes of intervention adaptation be described and systematically evaluated [132]. We are closely monitoring intervention delivery including the components of the intervention that were applied and the dose of the intervention that each participant actually received. Monitoring intervention implementation will provide data on the extent to which the intervention that was received deviates from the intervention as designed [127]. This information will be used to determine the key components and adaptable components of the intervention, thus facilitating future dissemination [132]. In addition, these data will be used to operationalize the intervention in the outcome analysis. That is, the intervention is represented by the actual dose received (number of home visits and case conferences). The outcome analysis consists of examining the relationship between the intervention dose and the level of fidelity to treatment and changes in the outcome scores from pre-test to post-test. This type of analysis accounts for variability in intervention implementation, which increases the statistical power to detect significant effects [133], facilitates a dose-response relationship, and enhances the clinical relevance of the study results [127]. The results will also allow informed speculation on the mechanism through which the intervention improves outcomes.

The third challenge centres on the need to determine the acceptability of the intervention or the willingness of clients and health care providers to adopt or use the intervention. Health promotion and disease prevention strategies need to be acceptable to the older adult receiving the intervention and the health care provider delivering the intervention. Thus, we will evaluate the experiences of the study participants and health care providers with the intervention, to determine the feasibility and acceptability of a wider implementation of the intervention. This will include providing empirical evidence on the facilitating and limiting factors that could influence effective use of the nurse-led intervention. We will also monitor the level of client adherence to the plan of care to determine the degree of uptake of the intervention.

The final challenge centres on the need to determine the sustainability of the intervention. This concerns whether or not the program will produce long-term behavioural change and whether organizations will sustain the program over time [126]. We will examine both the immediate 6-month and the 12-month effect of the intervention in order to provide information on the sustainability of the intervention effects over time. The results will also provide information on when the intervention becomes effective. The nurse-led intervention was designed and is delivered using existing home care services and supports. The organizations partnering in this research have demonstrated shared commitment for planning, implementation, and evaluation, shared vision and objectives, infrastructure support, stakeholder engagement and buy-in, and strong leadership support in the development of this nurse-led model. These are all essential factors that will contribute to the development of a practical, transferrable and sustainable practice model in this population. The research will build capacity in depression care and foster collaborative partnerships across the geriatric mental health care delivery system that will further enhance the sustainability of the intervention. Attending to these challenges may ultimately serve to enhance the relevance of the study results to clinicians and policy makers, and to help reduce the research-practice gap.

## Competing interests

The authors declare that they have no competing interests.

## Authors' contributions

All authors contributed to the design of this study. MFMR, CM, DF, MG, GB, and BB contributed to the design and implementation of the nurse-led intervention. MMR wrote the first draft of this manuscript, and all authors contributed to the discussion and editing. All authors read and approved the final manuscript. LT supervised the statistical analyses, JSH supervised the economic analyses, and all authors had full access to the data.

## Pre-publication history

The pre-publication history for this paper can be accessed here:

http://www.biomedcentral.com/1471-2318/11/50/prepub
